# *In vitro* and *in vivo* Characterization of Host–Pathogen Interactions of the L3881 *Candida albicans* Clinical Isolate

**DOI:** 10.3389/fmicb.2022.901442

**Published:** 2022-07-11

**Authors:** Pedro H. F. Sucupira, Tauany R. Moura, Isabella L. S. Gurgel, Tassia T. P. Pereira, Ana C. B. Padovan, Mauro M. Teixeira, Diana Bahia, Frederico M. Soriani

**Affiliations:** ^1^Centro de Pesquisa e Desenvolvimento de Fármacos, Laboratório de Genética Funcional, Departamento de Genética Ecologia e Evolução, Instituto de Ciências Biológicas, Universidade Federal de Minas Gerais, Belo Horizonte, Brazil; ^2^Departamento de Microbiologia e Imunologia, Universidade Federal de Alfenas, Alfenas, Brazil; ^3^Centro de Pesquisa e Desenvolvimento de Fármacos, Departamento de Bioquímica e Imunologia, Instituto de Ciências Biológicas, Universidade Federal de Minas Gerais, Belo Horizonte, Brazil; ^4^Departamento de Genética Ecologia e Evolução, Instituto de Ciências Biológicas, Universidade Federal de Minas Gerais, Belo Horizonte, Brazil

**Keywords:** *Candida albicans* infection, fungal infection, innate immunity, clinical isolates, virulence

## Abstract

*Candida albicans* is a human commensal fungus and the etiologic agent of nosocomial infections in immunocompromised individuals. *Candida* spp. is the most studied human fungal pathogen, and the mechanisms by which this fungus can evade the immune system affecting immunosuppressed individuals have been extensively studied. Most of these studies focus on different species of *Candida*, and there is much to be understood in virulence variability among lineages, specifically different *C. albicans* clinical isolates. To better understand the main mechanisms of its virulence variability modulated in *C. albicans* clinical isolates, we characterized L3881 lineage, which has been previously classified as hypovirulent, and SC5314 lineage, a virulent wild-type control, by using both *in vitro* and *in vivo* assays. Our findings demonstrated that L3881 presented higher capacity to avoid macrophage phagocytosis and higher resistance to oxidative stress than the wild type. These characteristics prevented higher mortality rates for L3881 in the animal model of candidiasis. Conversely, L3881 has been able to induce an upregulation of pro-inflammatory mediators both *in vitro* and *in vivo*. These results indicated that *in vitro* and *in vivo* functional characterizations are necessary for determination of virulence in different clinical isolates due to its modulation in the host–pathogen interactions.

## Introduction

*Candida albicans* is a human commensal fungus and the etiologic agent of infections in skin, oral and esophageal cavities, gastrointestinal tract, lungs, and bloodstream in immunocompromised individuals (Kadosh and Lopez-Ribot, 2013; Lemberg et al., 2022). Although it is considered an opportunistic fungus, *C. albicans* is the most common cause of nosocomial fungal infections, with a mortality rate of ∼40% in patients with systemic infections (Gudlaugsson et al., 2003; Perlroth et al., 2007; Erami et al., 2022; Jeong et al., 2022). Due to its severity, nosocomial candidemia contributes to prolonged hospitalization, increased healthcare costs, and increased morbidity and mortality rates (Perlroth et al., 2007; Arendrup, 2010; Kullberg and Arendrup, 2015; Erami et al., 2022; Jeong et al., 2022).

*Candida albicans* presents several biological characteristics that contribute to its pathogenicity (Calderone and Fonzi, 2001). The expression of adhesins and invasins contributes to an effective adherence and invasion of endothelial and epithelial cells, enabling their dissemination into the bloodstream. Adhesin and invasin also contribute to biofilm formation in implantable medical devices, which are the main source of long-term candidiasis (Schmidt et al., 2012; Desai et al., 2014; Sheppard and Filler, 2014; Kullberg and Arendrup, 2015; McCarty and Pappas, 2016). The secretion of aspartyl proteases and phospholipases promotes fungal tissue invasion and organ damage while simultaneously activating the innate immune response (Felk et al., 2002; Gabrielli et al., 2016), and the ability to alter their morphotypes among unicellular yeast cells—pseudohyphae, and hyphae favor the evasion of the immune system (Netea et al., 2006, 2015; Goodridge and Underhill, 2008; Wheeler et al., 2008; Marakalala et al., 2009; Gow et al., 2011). Finally, the ability to resist oxidative stress by inducing the expression of antioxidants such as catalase (*CAT1*), glutathione peroxidase (*GPX*), and superoxide dismutase (*SOD*) plays critical roles in repairing oxidatively damaged proteins (Enjalbert et al., 2003, 2006, 2007; Fradin et al., 2003, 2005; Rubin-Bejerano et al., 2003; Lorenz et al., 2004).

The mechanisms by which *Candida albicans* can evade the immune system affecting immunosuppressed individuals have been widely studied. However, there is much to be understood in variations among lineages, specifically different clinical isolates of *C. albicans*. Comparative studies among strains are important, given that intraspecific diversification is the first step in the evolutionary process, and the diversity among lineages may result in virulence differences and, more importantly, in their resistance to antifungal drugs.

L3881, isolated from a 44-year-old patient’s bloodstream, has achieved its hypovirulent status based on the presence of a mutation in *HWP1* gene, which reduces the biofilm production and hyphal formation in the homozygous lineage (Padovan et al., 2009) – referred to as L757 strain. Conversely, a study carried out by Maza et al. (2017) showed, *in vitro*, that L3881 presented higher resistance to oxidative stress the wild-type SC5314, a characteristic that may be correlated to higher virulence (Maza et al., 2017). Therefore, there is no adequate experimental evidence to consider L3881 hypovirulent.

To better understand the main mechanisms modulated in *C. albicans* clinical isolates that lead to changes in its virulence ability, we analyzed both L3881 and SC5314 *in vitro* and *in vivo* behaviors. Overall, our findings demonstrated that L3881 was less phagocytized by macrophages and presented higher resistance to oxidative stress than wild-type SC5314. These characteristics prevented higher mortality rates due to L3881 infection in animal models of candidiasis. However, L3881 induced an upregulation of pro-inflammatory mediators both *in vitro* and *in vivo*. These data indicated that *in vitro* and *in vivo* functional characterizations are necessary for virulence determination in different clinical isolates as a response to modulation of the host–pathogen interactions.

## Materials and Methods

### Ethics Statement

All animal experiments were approved by the Institution Ethics Committee (Comitê de Ética em Experimentação Animal, CETEA/UFMG, Protocol number 62/2011) and were conducted in accordance with Brazilian national guidelines on animal work (Conselho Nacional de Controle de Experimentação Animal – CONCEA).

### *Candida albicans* Lineages and Culture Conditions

All experiments were carried out using two lineages of *C. albicans*: L3881, initially referred to as L757 lineage, which was isolated from the blood of a 44-year-old patient who had candidemia and was cured clinically (Padovan et al., 2009), and SC5314 lineage, which was first isolated in 1984 from a patient with disseminated candidiasis and has been widely used as a reference lineage (Gillum et al., 1984). The isolates were kindly provided by LEMI (Laboratório Especial de Micologia, Universidade Federal de São Paulo, Brazil). *C. albicans* isolates were grown overnight (16 h) in the YPD medium (1% [w/v] yeast extract; 2% [w/v] peptone, and 2% [w/v] dextrose) at 30°C. A single colony of *C. albicans* isolates was inoculated in the YPD liquid medium and incubated in an orbital shaker at 30°C overnight. Then, *C. albicans* cells were washed with phosphate-buffered saline (PBS) – pH 7.2, harvested by centrifugation, and counted in a hemocytometer chamber.

### Animal Infections and Tissue Analysis

For this study, we used 8- to 10-week-old male mice BALB/c, obtained from Centro de Bioterismo (UFMG/Brazil). The mice were maintained in pathogen-free air-conditioned microisolators with an enriched environment at Laboratório de Imunofarmacologia (UFMG/Brazil) and supplied with mouse chow and filtered water *ad libitum*. Prior to infection, the mice were divided in three groups: control, L3881, and SC5314. All the mice experiments were double-blinded and randomly assigned. The animal experiments were carried out using at least five animals. The survival curves were plotted using at least 10 animals per group. The mice in the control group received saline solution intravenously by the tail, while the mice in L3881 and SC5314 groups were infected with 2.5 × 10^4^ colony-forming units (CFUs)/kg of *C. albicans* L3881 and SC5314 lineages, respectively, in a total volume of 100 μL of sterile PBS. At 2 days post-inoculation, the mice in the three groups were euthanized with a solution of 180 mg/kg of ketamine and 24 mg/kg of xylazine. Subsequently, blood was harvested from the tail vein, and lungs, liver, and kidney were harvested and immediately frozen in liquid nitrogen for subsequent analysis of myeloperoxidase (MPO) (Huang et al., 2016) and N-acetylglucosaminidase (NAG) (Reiner et al., 1981). Quantification of NAG and MPO shows the relative number of macrophages and neutrophils, respectively. Exclusively, lungs were perfused with 5 mL of PBS to remove circulating blood, and the right lobes were removed for MPO and NAG analysis. Blood and homogenates from the lungs, liver, and kidney were plated on YPD-agar, and CFUs were determined after overnight incubation at 30°C.

### Cytokine and Chemokine Measurement

Cytokine and chemokine (TNF-α, IL-10, IL-4, IFN-γ, CXCL1, and CCL2) levels were quantified using DuoSet ELISA kits (R&D Systems) in accordance with the manufacturer’s protocol.

### Cell Culture

The murine macrophage RAW 264.7 cell line was obtained from the American Type Culture Collection and cultivated as described in Souza et al. (2019). Briefly, the cells were incubated in Dulbecco’s minimal essential medium (DMEM), supplemented with fetal bovine serum (FBS) 10% v/v and 2 mM L-glutamine. The cells were maintained at 37°C in a 5% CO_2_ atmosphere and were incubated in 24-well plates (1 × 10^6^ cells/well) with L3881 and SC5314 lineages (5 × 10^6^ yeast/well), multiplicity of infection (MOI) 5:1, for 2 and 4 h. After the incubation period, the supernatant was collected for cytokine and chemokine quantification.

### Phagocytosis and Killing Assays

Phagocytosis and killing assays were conducted as previously described (Malacco et al., 2020). Briefly, RAW 264.7 cells (1 × 10^5^ cells/well) were incubated with L3881 or SC5314 lineage (3 × 10^5^ yeast/well), MOI 3:1, at 37°C. Phagocytosis was evaluated in cytospin preparations. After the incubation period of 4 h, the cells were stained using a Quick Panoptic kit (Laborclin), and phagocytosis was evaluated under an optical microscope. The phagocytic index was calculated by multiplying the percentage of macrophages that had one or more phagocytized yeast by the ratio of the total number of cells per well to the number of cells that phagocytized.

Killing was assessed by CFU counting. After incubating for 6 h, macrophages were washed with 1× PBS to remove the non-phagocytized yeasts and lysed with 200 μL of water for injection added in each well. Subsequently, diluted samples were plated in the YPD medium, and CFUs were determined after 16 h of incubation at 30°C. The intracellular proliferation rate was calculated as CFUs/PI, where PI represents the phagocytic index.

### Sensitivity to Reactive Oxygen Species

To measure the sensitivity of *C. albicans* to ROS, 3 × 10^5^ yeast/mL of each lineage was incubated in the YPD medium in an orbital shaker at 30°C for 1 h with three different H_2_O_2_ concentrations (5 mM, 10 mM, and 20 mM). Afterward, *C. albicans* cells were washed with PBS, harvested by centrifugation, plated on the YPD medium. CFUs were determined after 16 h of incubation at 30°C.

### Lipid Peroxidation

Lipid peroxidation was measured using an assay based on the reaction of lipid peroxides with thiobarbituric acid as, described by Hermes-Lima et al. (1995). For this experiment, yeast and hyphae of L3881 and SC5314 lineages were used. To obtain hyphae, C. albicans lineages were incubated in a liquid complete medium (YPD) supplemented with 10% fetal bovine serum (FBS) at 37°C for 1 h. Briefly, yeast and hyphae from L3881 and SC5314 lineages (1 × 10^9^ yeast cells) were mixed with 200 μL of a cold solution containing 1% TBA (thiobarbituric acid), 0.05 M NaOH, and 0.1 mM butylated hydroxytoluene (BHT) plus phosphoric acid 7%. The samples were heated for 15 min at 100°C, and 750 μL of butanol was added. The samples were shaken for 10 s and centrifuged for 5 min at 800 *g*. The organic layer was removed, and absorbances at 532 and 600 nm were measured. For the blanks, TBA solution was replaced by 3 mM HCI. We used the molar extinction coefficient for MDA (malondialdehyde – final product of lipid peroxidation) in the denominator to calculate nanomoles of TBARS. TBARS results were calculated, and values were expressed as nmol.g^–1^.


$$TBARS(nmol.g^{-1})=\{[Absorbance_{sample}(A532-A600)-Absorbance_{blank}(A532-A600)]\times {\mathrm{10}}^{3}\}/156$$

### Antioxidant Enzyme Assays

Glutathione (GSH) was measured using the enzymatic recycling procedure by Tietze (1969), in which GSH is sequentially oxidized by Ellman’s reagent {DTNB [5,5-dithio-bis-(2-nitrobenzoic acid)]} and reduced to NADPH in the presence of glutathione reductase (Tietze, 1969). Briefly, *C. albicans* cell suspensions were macerated with liquid NO_2_, resuspended in 0.4 M Tris–HCl buffer – pH 8.9, and then precipitated with 12.5% trichloroacetic acid (TCA). The resulting cell homogenate was centrifuged at 10,000 × *g* for 15 min at 4°C. The supernatant was mixed with HCl buffer + DTNB and then used to measure the antioxidant enzyme activities. The protein concentration was measured at 495 nm using the Bradford assay (1976) with bovine serum albumin as the standard (Bradford, 1976).

Catalase activity was measured as described by Aebi (1984) by following the decrease in absorbance of H_2_O_2_ (Aebi, 1984). Briefly, *C. albicans* cell suspensions were macerated with liquid NO_2_ and then resuspended in 0.05 M phosphate buffer – pH 7. To measure the absorbance, 20 μL of the cell homogenate was mixed with 980 μL of reaction buffer (H_2_O_2_ + 0.005 M phosphate buffer – pH 7). The decrease in absorbance was measured every 10 s for 1 min at 240 nm. Standards containing 5, 10, and 20 mM of H_2_O_2_ were used to construct a standard curve.

### Statistical Analyses

Data analysis was performed using GraphPad Prism v.9.1.1 software (San Diego, CA, United States). The Shapiro–Wilk normality test was conducted to assess data normality distribution. Considering the parametric distribution of the results, comparative analysis between two groups was performed by using the unpaired *t*-test. Multiple comparative analysis among subgroups was carried out by using one-way analysis of variance (ANOVA), followed by Tukey’s *post hoc* test. In all cases, statistical significance was set as *p* < 0.05. Data are presented as mean ± standard deviation (SD). Survival analysis was made by using the log-rank test.

## Results

### L3881 Is Less Phagocytized by Macrophages and Presented a Higher Survival Rate After Phagocytosis

**FIGURE 1:**
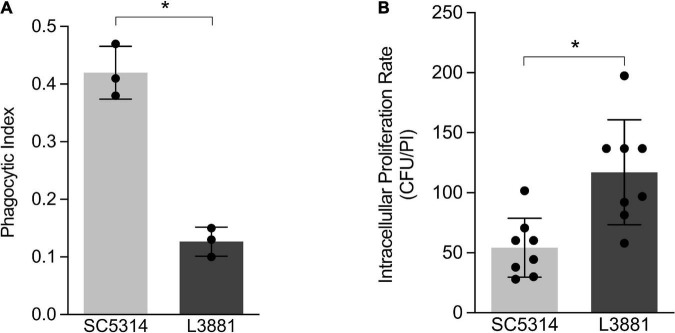
*In vitro* characterization of *Candida albicans* SC5314 and L3881 lineages. **(A)** Phagocytosis was assessed by cytospin preparations of murine macrophage RAW 264.7 cell line co-cultured with SC5314 and L3881. **(B)** Killing assay was evaluated by cell lysis with water, the diluted samples were plated in the fungal medium, and colony-forming units (CFUs) were determined 16 h post-incubation (PI). Each dot represents an experimental replica. Data are presented as mean ± standard deviation (SD). Asterisk (*) represents significant differences with *p* < 0.05.

Aiming at characterizing the role of macrophages against SC5314 and L3881 *C. albicans* lineages, we analyzed the phagocytosis rate using *in vitro* assays with murine macrophage RAW 264.7 cell line. Data analysis demonstrated that L3881 was 2.5 times less phagocytized than the wild-type strain (Figure 1A). Additionally, we analyzed the viable fungal cells after phagocytosis. We observed that L3881 displayed a higher proliferation rate after been phagocytized than SC5314 (Figure 1B). The growth of both strains was evaluated, and there was no difference in growth between SC5314 and L3881 (Supplementary Figure 1).

### L3881 Showed Higher Oxidative Stress Tolerance

**FIGURE 2:**
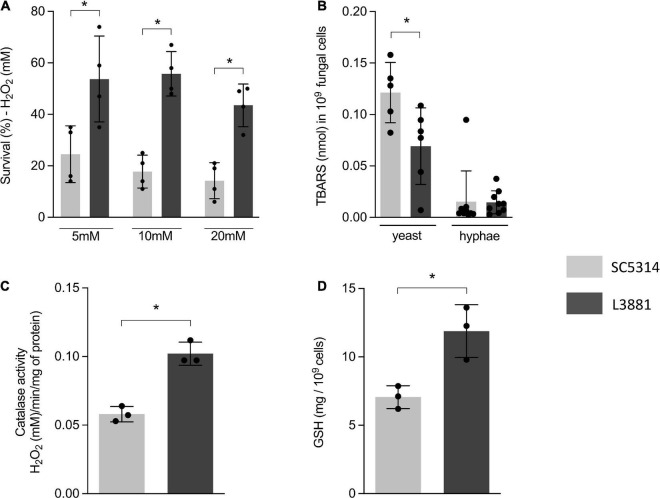
Antioxidant activity of SC5314 and L3881 lineages. **(A)** *C. albicans* yeast cells of SC5314 and L3881 lineages were incubated with three different concentrations of H_2_O_2_ (5, 10, and 20 mM). **(B)** Lipid peroxidation was measured by the formation of products of oxidation of thiobarbituric acid. **(C)** Catalase activity was measured by following the absorbance decrease in H_2_O_2_. **(D)** Glutathione (GSH) was quantified using the enzymatic recycling procedure, reducing NAD^+^ to NADPH in the presence of glutathione reductase. Each dot represents an experimental replica. Data are presented as mean ± SD. Asterisk (*) represents significant differences with *p* < 0.05.

The higher capacity of L3881 to proliferate intracellularly within macrophages, as demonstrated in Figure 1B, was very intriguing. Therefore, aiming at further characterizing the dynamic of its antioxidant activity, we exposed both lineages to three different concentrations of H_2_O_2_ (5, 10, and 20 mM). The results showed that L3881 was more resistant to H_2_O_2_, with a survival rate two times the wild-type control, SC5314, even at higher concentrations of H_2_O_2_ (Figure 2A).

To further characterize the antioxidant activity of L3881 and SC5314, we assessed lipid peroxidation levels by TBAR assay to evaluate oxidative damage, both in the yeast and hyphal forms. We found that L3881 significantly underwent a less effect of lipid peroxidation when exposed to H_2_O_2_ in the yeast form than SC5314 (Figure 2B). Moreover, we quantified catalase (CAT) activity and glutathione (GSH) levels. The results showed that L3881 lineage presented two times more of these antioxidant systems (catalase – Figure 2C and reduced glutathione – Figure 2D) than the wild type.

### L3881 Elicited Lower Levels of Pro-inflammatory Mediators *in vitro*

**FIGURE 3:**
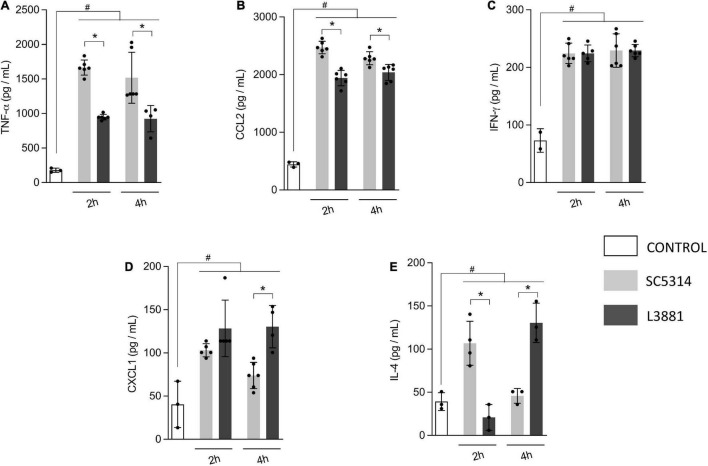
*In vitro* cytokine and chemokine profile of L3881 and SC5314. Murine macrophage RAW 264.7 cell line was infected with L3881 and SC5314 yeast lineages. Supernatants were collected at 2 and 4 h post-incubation and used for ELISA for quantifications of **(A)** TNF-α, **(B)** CCL2, **(C)** IFN-γ, **(D)** CXCL1, and **(E)** IL-4 levels. Each dot represents an experimental replica. Data are presented as mean ± SD. Hashtag (#) and asterisk (*) represent significant differences with *p* < 0.05.

Aiming to further characterize macrophage response to both *C. albicans* lineages, we quantified the levels of inflammatory mediators produced by murine macrophage RAW 264.7 cell line co-cultured with yeasts for 2 and 4 h. Data analysis demonstrated that macrophages infected with L3881 showed a significant decrease in TNF-α and CCL2 levels at both time points, as compared to cells infected with SC5314 (Figures 3A,B). Conversely, CXCL1 levels were significantly increased in L3881-infected cells at 4 h post-infection (h.p.i) (Figure 3D). Interestingly, for IL-4 levels, the profiles displayed were inversely proportional. While cells infected with L3881 displayed a progressive increase over time, SC5314-infected cells displayed a progressive decrease (Figure 3E). There was no significant difference in IFN-γ levels (Figure 3C).

### L3381 Was Able to Spread and Infect Tissues

**FIGURE 4:**
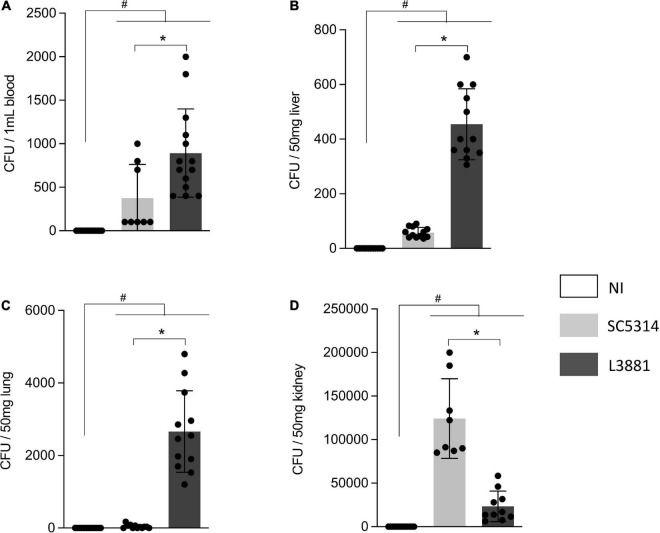
CFU quantification in mice infected with *C. albicans* lineages. BALB/c male mice were infected with SC5314 or L3881 intravenously through the tail vein. Mice in the non-infected group (NI) received saline solution. At 2 days post-inoculation, the blood, liver, lungs, and kidney were collected. CFUs were determined for **(A)** blood and homogenates from the **(B)** liver, **(C)** lung, and **(D)** kidney. Each dot represents tissue from an individual mouse. Data are presented as mean ± SD. Hashtag (#) and asterisk (*) represent significant differences with *p* < 0.05.

We investigated the virulence of the two lineages, L3881 and SC5314, by measuring their ability to spread and invade different tissues in animal models of candidiasis. Our results showed that both lineages were able to spread through the bloodstream. However, L3881 proliferated into the bloodstream, presenting a higher number of CFUs than SC5314 (Figure 4A). This difference was also seen in the liver and lungs, where L3881 presented a higher number of CFUs (Figures 4B,C). On the other hand, in the kidney, despite invasiveness of both lineages, SC5314 presented a higher number of CFUs (Figure 4D).

### L3881 Recruited More Macrophages and Neutrophils Into the Site of Infection *in vivo*

After confirming that both lineages were able to invade specific tissues, we investigated how microbiological characteristics of each lineage were able to sensitize the immune system and trigger a proper immune response to infection. This was initially achieved by analyzing macrophage and neutrophil migration to tissues infected with *Candida*.

**FIGURE 5:**
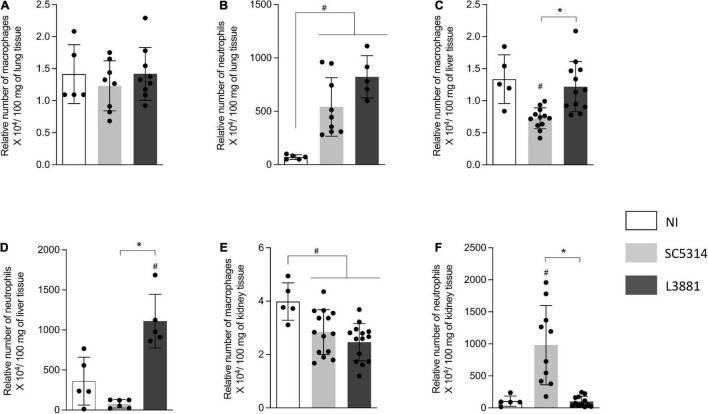
Relative number of macrophages and neutrophils in the lung, liver, and kidney from mice infected with *C. albicans* lineages BALB/c male mice were infected with SC5314 or L3881 intravenously through the tail vein. Mice in the non-infected group (NI) received saline solution. At 2 days post-inoculation, the liver, lungs, and kidney were collected for NAG (relative number of macrophages) and MPO (relative number of neutrophils) quantification. **(A)** NAG in the lung. **(B)** MPO in the lung. **(C)** NAG in the liver. **(D)** MPO in the liver. **(E)** NAG in the kidney. **(F)** MPO in the kidney. Each dot represents tissue from an individual mouse. Data are presented as mean ± SD. Hashtag (#) and asterisk (*) represent significant differences with *p* < 0.05.

Into the lungs, there was no difference in macrophage recruitment between the groups (Figure 5A). However, the relative number of neutrophils was higher in the mice infected with *C. albicans*, regardless of the lineage, than in the reference non-infected mice (NI) (Figure 5B).

In the liver, the mice infected with SC5314 recruited less macrophages than other groups (Figure 5C). Of note was the outstanding increase in neutrophils recruited only in the mice infected with L3881 (Figure 5D).

L3881 and SC5314 showed an effect on the relative number of macrophages in the kidney. The mice infected with SC5314 or L3881 presented a lower number of macrophages than NI (Figure 5E). On the other hand, the relative number of neutrophils was only affected by infection with SC5314 (Figure 5F).

### L3881 Disrupted Immune Response Despite Immune Cell Recruitment

Since L3881 and SC5314 were able to invade the lung, liver, and kidney, we expected that the activation of immune response was triggered through the production of cytokines and chemokines. Therefore, we quantified the levels TNF-α, IL-10, CXCL1, IL-4, and INF-γ into the lungs, liver, and kidney.

**FIGURE 6:**
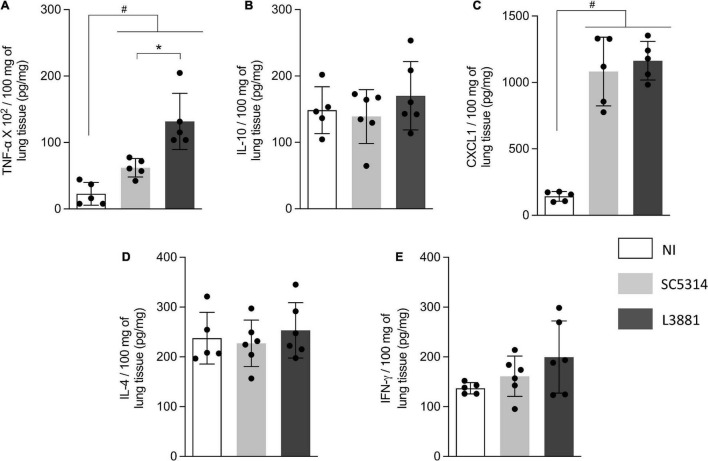
Cytokine and chemokine profiles of lung tissue from mice infected with *C. albicans* lineages. BALB/c male mice were infected with SC5314 or L3881 intravenously through the tail vein. Mice in the non-infected group (NI) received saline solution. At 2 days post-inoculation, lungs were collected for **(A)** TNF-α, **(B)** IL-10, **(C)** CXCL1, **(D)** IL-4, and **(E)** INF-γ by ELISA. Each dot represents tissue from an individual mouse. Data are presented as mean ± SD. Hashtag (#) and asterisk (*) represent significant differences with *p* < 0.05.

In the lungs, data analysis demonstrated that regardless of the lineage, the mice infected with *C. albicans* displayed a higher production of TNF-α and CXCL1 (Figures 6A,C) than NI, with a higher upregulation of TNF-α on L3881-infected mice. There were no significant differences between the expression levels of INF-γ, IL-10, and IL-4 (Figures 6B,D,E).

**FIGURE 7:**
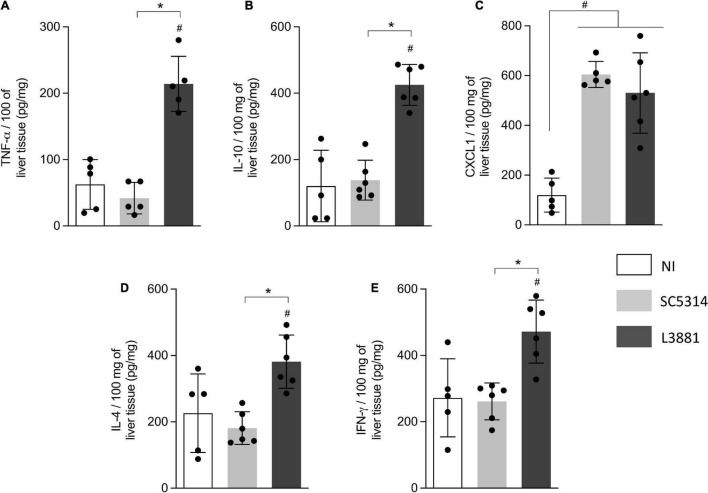
Cytokine and chemokine profiles of liver tissue from mice infected with *C. albicans* lineages. BALB/c male mice were infected with SC5314 or L3881 intravenously through the tail vein. Mice in the non-infected group (NI) received saline solution. At 2 days post-inoculation, the liver as collected for **(A)** TNF-α, **(B)** IL10, **(C)** CXCL1, **(D)** IL4, and **(E)** INF-γ by ELISA. Each dot represents tissue from an individual mouse. Statistical analyses were performed using the unpaired *t*-test. Data are presented as mean ± SD. Hashtag (#) and asterisk (*) represent significant differences with *p* < 0.05.

In the liver, the levels of TNF-α, IL-10, IL-4, and INF-γ (Figures 7A,B,D,E) were significantly higher in the mice infected with L3881 than those in the SC5314-infected mice and NI. CXCL1 levels were increased in both SC5314 and L3881 as compared to NI (Figure 7C).

**FIGURE 8:**
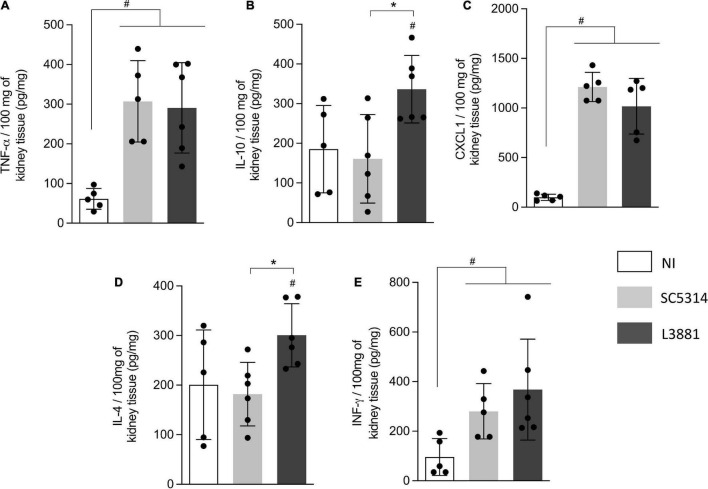
Cytokine and chemokine profiles of kidney tissue from mice infected with *C. albicans* lineages. BALB/c male mice were infected with SC5314 or L3881 intravenously through the tail vein. Mice in the non-infected group (NI) received saline solution. At 2 days post-inoculation, kidneys were collected for **(A)** TNF-α, **(B)** IL10, **(C)** CXCL1, **(D)** IL4, and **(E)** INF-γ by ELISA. Each dot represents tissue from an individual mouse. Statistical analyses were performed using the unpaired *t*-test. Data are presented as mean ± SD. Hashtag (#) and asterisk (*) represent significant differences with *p* < 0.05.

In the kidney, the levels of TNF-α, INF-γ, and CXCL1 were significant higher in both groups of *C. albicans*-infected mice than those in NI (Figures 8A,C,E), while the levels of IL-10 and IL-4 were significantly higher in the mice infected with L3881 than those in SC5314 and NI (Figures 8B,D).

### L3881-Infected Mice Presented Higher Mortality

**FIGURE 9:**
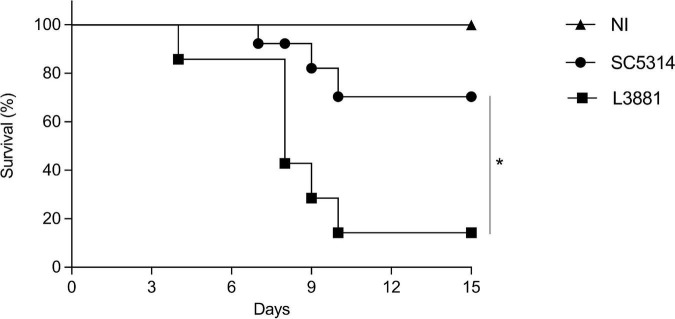
Mortality rate of mice infected with *C. albicans* lineages. BALB/c male mice were infected with SC5314 or L3881 intravenously through the tail vein, and survival was monitored for 15 days. Mice in the non-infected group (NI) received saline solution. Comparative lethality curves of NI and infected groups were performed. Data are presented as mean ± SD. Asterisk (*) represents significant differences with *p* < 0.05.

We next assessed the mortality rate of the mice infected with L3881 or SC5314. BALB/c male mice were infected with each of the lineages, and their survival rates were monitored for 15 days. As shown in Figure 9, 20% of the mice infected with L3881 died at 3 days post-infection (d.p.i), while the mice infected with SC5314 reached 20% lethality only at 9 d.p.i. The highest mortality rate was observed at 10 d.p.i when 90% of the L3881-infected animals succumbed, while animals infected with the wild-type strain presented a survival rate of 70% at the same time point.

## Discussion

*Candida albicans* is the most studied human commensal fungus and the most common etiologic agent of nosocomial fungal infections. As an opportunistic fungus, the ability of *C. albicans* to cause disease is due to its capacity to invade, evade the immune system, and infect different organs. The diversity of clinical isolates, in terms of virulence, makes the understanding of the biological/genetic factors are responsible for their virulence more complex. In this sense, using different clinical isolates, several studies have assessed the biological features of *C. albicans* that may serve as virulence factors (Reviewed by Dadar et al., 2018).

SC5314 has been widely acknowledged as a virulent lineage in mouse models, and since its isolation, it has been used as a wild-type reference in previous studies (Padovan et al., 2009; Lionakis et al., 2011; Maza et al., 2017; Shankar et al., 2020; Lemberg et al., 2022). In turn, the studies that employed L3881 (first described as L757 lineage, Padovan et al., 2009) have not yet reached a conclusion about its virulence status.

A study carried out by Padovan and colleagues characterized L3881 as hypovirulent based on hyphal wall protein 1 (*HWP1*) gene. This gene is responsible for encoding a cell surface protein expressed only in hyphae, whose main function is to bind to epithelial cells. L3881 was characterized as a homozygous *HWP1-2* lineage that showed reduced *HWP1* expression, lower biofilm formation levels, and defective hyphae development (Padovan et al., 2009). In another study carried out by Maza, Bonfim-Melo and colleagues, in addition to producing shorter hyphae, L3881 showed lower internalization into HeLa cells and damage than the SC5314 lineage, but conversely was more resistant to oxidative stress (Maza et al., 2017). Despite genetic factors, the ability to cause disease is strongly related to the host–pathogen relationship outcomes, as discussed in Bahia et al. (2018). Therefore, aiming at overcoming these previous discrepancies, we have performed a detailed analysis of the main mechanisms that enable *C. albicans* SC5314 and L3881 lineages to cause disease.

To investigate host–pathogen interactions, we analyzed both *in vitro* and *in vivo* behavior of these lineages. Tissue-residing macrophages are the first innate immune cells that are key effector in antifungal defense, and previous studies have demonstrated the relevance of macrophages in anti-*C. albicans* host defense. In a study by Qian et al. (1994), macrophage-depleted mice showed accelerated fungal proliferation in tissues and increased mortality (Qian et al., 1994). Here, we showed that the L3881 lineage was better able to face the macrophage antifungal defense *in vitro* given that it was less phagocytized and displayed higher proliferation rate after been phagocytized than the reference lineage SC5314.

After phagocytosis, respiratory burst with ROS production is an important antimicrobial defense mechanism. The ROS generated within the phagosome creates a toxic environment that induces oxidative stress in *C. albicans*, leading to irreversible damage. Previous studies have demonstrated that *C. albicans* is resistant to ROS and has a well-known ability to induce an antioxidative response to phagocytosis by secreting a range of antioxidants, such as catalase (CAT) and glutathione (GSH) (Enjalbert et al., 2003, 2006, 2007; Fradin et al., 2003, 2005; Rubin-Bejerano et al., 2003; Lorenz et al., 2004; Brown et al., 2014).

Our data demonstrated that in addition to better dealing with macrophage phagocytosis, L3881 was more resistant to oxidative stress, in agreement with Maza, Bonfim-Melo and colleagues (Maza et al., 2017). In this study, we demonstrated, for the first time, some mechanisms responsible for this resistant phenotype: L3881 showed higher CAT and GSH enzymatic activity creating a more efficient detoxification of host-derived ROS than SC5314.

Subsequently, we evaluated the cytokine profile secreted by macrophages upon fungi invasion. L3881 induced lower secretion of TNF-α whose pro-inflammatory effects may have been modulated by the progressive increase in IL-4. Rocha et al. (2017) demonstrated that TNF-α can prevent *in vitro* biofilm development of *C. albicans* yeast forms (Rocha et al., 2017).

Taken together, we hypothesized that L3881 takes advantage of macrophage activity *in vitro* due to its persistence against phagocytosis, higher resistance to oxidative stress, and an altered stimulation of these cells, generating distinct profiles of the secreted cytokines analyzed, suggesting that L3881 has a virulent potential. To understand deeper about L3881 ability to cause disease, we analyzed it in an *in vivo* model of candidemia. The pathogenesis of *C. albicans* infection involves a complex interaction between fungal and host features. The properties that are central to *C. albicans* pathogenesis are as follows: first, the ability to morphologically switch between yeast and hyphal forms and, second, biofilm formation, both allowing this pathogen to invade, spread, and infect tissues (Jabra-Rizk et al., 2004; Ramage et al., 2005; Calderone, 2012; Chauvel et al., 2012). Although L3881 presented *HWP1* natural deletion and shorter hyphae, our data demonstrated that both lineages were able to spread through the bloodstream and invade the liver, lungs, and kidney of infected mice, confirming their virulent status. Emphasis is placed on the fact that L3881 presented higher CFU counts in the blood, liver, and lungs, while the kidney was the only organ in which SC5314 presented higher CFU counts. The SC5314 infection profile in the kidneys was consistent with previously published data (MacCallum et al., 2009; Lionakis et al., 2011; Lüttich et al., 2013). Lüttich and colleagues showed that the SC5314 lineage can persist in the kidney over time, even in animals showing no clinical symptoms (Lüttich et al., 2013). Similarly, other studies have shown that the kidney is a common target of disseminated *Candida* infection in mice (Louria, 1985; Papadimitriou and Ashman, 1986).

The outcome of *C. albicans* infection is also related to host immune response, and we assessed this by quantifying immune cells and inflammatory mediators (Ashman, 2008; Netea et al., 2008). In this study, for the first time, we showed the profile of macrophages and neutrophils recruited and of inflammatory mediators secreted in the L3881-infected mice.

Upon increased fungal load or breach of defensive tissue barriers, macrophages and neutrophils are recruited to infection sites where they kill *C. albicans* by a combination of mechanisms including phagocytosis, degranulation to release toxic mediators, and ROS production.

Our data demonstrated that neutrophils are the most recruited immune defense cells, regardless of the tissue. Despite the large number of phagocytic cells recruited, L3881 was still able to infect all tissues much more robustly than SC5314. This could be explained by the higher capacity of L3881 to face phagocytosis and its antioxidant system. Moreover, previous studies have shown that the SC5314 lineage is very susceptible to phagocytosis by neutrophils (Shankar et al., 2020). However, there was a higher fungal load in the kidneys, despite the increase in neutrophil recruitment.

The cytokine levels showed variations among lineages and organs. In this sense, distinct *C. albicans* lineages that lead to diverse immune responses may impact the disease outcome toward severe clinical forms.

Overall, the SC5314-infected mice displayed a milder and less complex immune response than the L3881-infected mice. In the latter, it is evident that there is a loss of control of the inflammatory immune response in the liver, with higher levels of pro- and anti-inflammatory mediators, and accumulation of neutrophils that are unable to efficiently eliminate fungi from the liver. Off note, regardless of the lineage and organ, all infected mice presented increased CXCL1 levels, which may explain higher recruitment of neutrophils, an effect potentiated by the high levels of TNF-α – the most predominant pro-inflammatory cytokine.

TNF-α is a macrophage-derived cytokine which can enhance the antimicrobial activity of neutrophils (Djeu et al., 1986; Cybulsky et al., 1988). Previous studies have already shown the protective effect of TNF-α during candidiasis. Riipi and Carlson (1990) observed that TNF-α plays a crucial role *in vivo* in response to disseminated *Candida*. The treatment of mice with monoclonal antibodies against TNF-α enhanced the mortality to experimental disseminated candidiasis (Steinshamn and Waage, 1992; Louie et al., 1994). Likewise, treatment with pharmacological inhibitors of TNF-α production led to enhanced mortality and increased outgrowth of *C. albicans* during disseminated candidiasis in mice (Netea et al., 1995). Knock-out mice for TNF-α and β were highly susceptible to disseminated candidiasis (Marino et al., 1997).

However, TNF-α works like a double-edged sword. The excessive release of TNF-α during inflammation can become damaging, thus leading to cell infiltration, hypotension, hepatotoxicity, and structural damage to tissues (Streetz et al., 2000; Kalliolias and Ivashkiv, 2016). The high tissue invasiveness and colonization capacity of L3881 together with the increased amount of TNF-α induced by this lineage may lead to premature death. Therefore, to confirm this result, we evaluated the mortality rate caused by each of the lineages in mice. At the end of 15 days, only 10% of the L3881-infected mice survived compared to 70% of the SC5314-infected mice.

Our results have shown that the ability to better deal with the immune system *via* resistance to oxidative stress, to invade different tissues, and to interfere with the immune response confirmed the classification of L3881 as a virulent lineage, despite its short hyphae. We raised the discussion that the determination of *C. albicans* virulence should not only be based only on genotypic characterization but also on the host–pathogen interaction outcomes. Furthermore, the virulence of a lineage is also related to the infection model used. Recently, Dunker et al. (2021) have demonstrated that a filament-deficient lineage showed different virulence profiles depending on the infection model used. In this study, the same lineage generated an attenuated infection in an intraperitoneal infection model, whereas in a systemic infection model, it was as virulent as a wild type (Dunker et al., 2021). This study demonstrated how the determination of the virulence status of a given lineage depends on several factors and cannot be determined considering only one feature.

## Data Availability Statement

The raw data supporting the conclusions of this article will be made available by the authors, without undue reservation.

## Ethics Statement

The animal study was reviewed and approved by the Institutional Ethics Committee (Comitê de Ética em Experimentação Animal, CETEA/UFMG, Protocol number 06/2011).

## Author Contributions

TM, DB, and FS: designing research study. MT and FS: funding acquisition. PS, IG, and TM: conducting experiments and acquiring data. PS, IG, TM, AP, TP, and FS: analyzing data. PS, IG, DB, and FS: writing and reviewing the manuscript. All authors contributed to the article and approved the submitted version.

## Conflict of Interest

The authors declare that the research was conducted in the absence of any commercial or financial relationships that could be construed as a potential conflict of interest.

## Publisher’s Note

All claims expressed in this article are solely those of the authors and do not necessarily represent those of their affiliated organizations, or those of the publisher, the editors and the reviewers. Any product that may be evaluated in this article, or claim that may be made by its manufacturer, is not guaranteed or endorsed by the publisher.
