# Supplementary material for: In vitro and in vivo Characterization of Host–Pathogen Interactions of the L3881 Candida albicans Clinical Isolate
Source: Front Microbiol. 2022 Jul 11;13:901442. doi: 10.3389/fmicb.2022.901442 (PMC9309619; doi:10.3389/fmicb.2022.901442)
Supplement: Supplementary file 1 [file Data_Sheet_1.pdf]

***In vitro* and *in vivo* characterization of host-pathogen interactions of the L3881 *Candida albicans* clinical isolate.**

Pedro H. F. Sucupira[1], Tauany R. Moura[1], Isabella L. S. Gurgel[1], Tassia Pontes[1], Ana C. B. Padovan[2], Mauro M. Teixeira[3], Diana Bahia[4], Frederico M. Soriani[1]

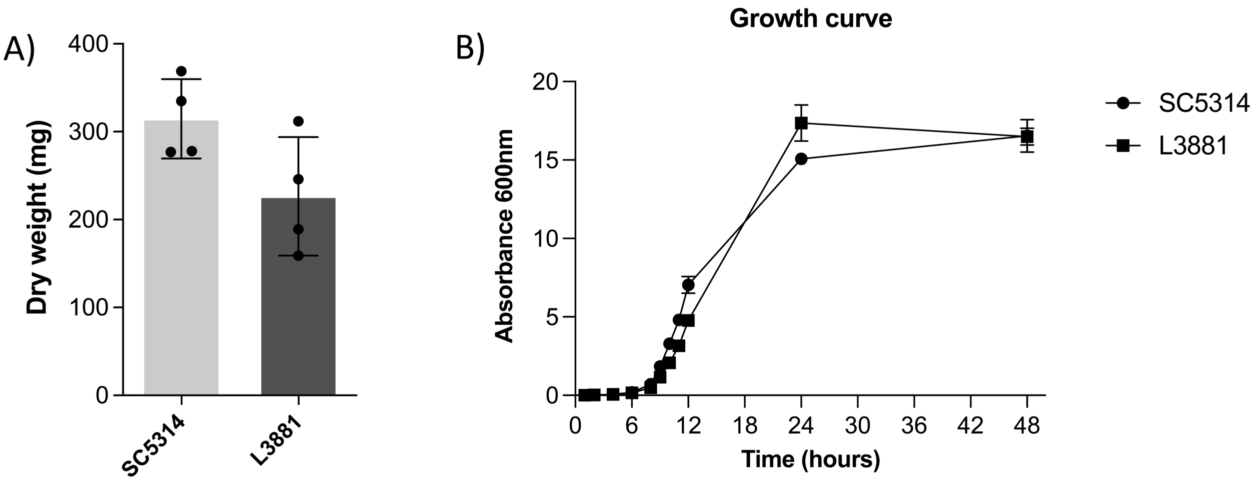

Supplementary Figure 1 – Dry weight and growth curve of SC5314 and L3881 *Candida albicans* lineages. **A)** Dry weight of SC5314 and L3881 lineages were measured after overnight growth in YPD medium at 30ºC. Each dot represents an experimental replica. Data are presented as the mean ± standard deviation (SD). Asterisk (*) represents significant differences with p<0.05. **B)** Growth curves of SC5314 and L3881 lineages were measured every 6 hours for 48 hours. The experiment was performed in quadruplicate. Data are presented as the mean ± standard deviation (SD) of the weight of all experimental replicates. Asterisk (*) represents significant differences with p<0.05.

Supplementary Materials and methods

C. albicans strains were grown in YPD medium (2% peptone, 1% yeast extract and 2% dextrose) at 30ºC in a shaking incubator. The strains were grown overnight in 10 ml of YPD at 30 ºC, then cells were harvested in 50 ml of YPD at an initial concentration of 1×10^6 cells/ml.

For dry weight measurement, cells were cultured for 24 h, then cultures were added to pre-weighed 50 mL falcon tubes and centrifuged at 2700 rpm for 10 minutes. Supernatant was discarded and the tubes were let dry at 56 ºC overnight. After that, pre-weighted tubes containing the dried pellet were weighted (mg) and the biomass was determined. The experiment was performed in quadruplicate.

To construct the growth curves, cells were cultured for 48 h. About 1 mL of the culture was collected at different time points. Absorbance was measured at 600 nm. The experiment was performed in quadruplicate.
